# Emotion recognition using Kinect motion capture data of human gaits

**DOI:** 10.7717/peerj.2364

**Published:** 2016-09-15

**Authors:** Shun Li, Liqing Cui, Changye Zhu, Baobin Li, Nan Zhao, Tingshao Zhu

**Affiliations:** 1 Institute of Psychology, Chinese Academy of Sciences, Beijing, China; 2 The 6th Research Institute of China Electronics Corporation, Beijing, China; 3 School of Computer and Control, University of Chinese Academy of Sciences, Beijing, China

**Keywords:** Emotion recognition, Affective computing, Gait, Machine learning, Kinect

## Abstract

Automatic emotion recognition is of great value in many applications, however, to fully display the application value of emotion recognition, more portable, non-intrusive, inexpensive technologies need to be developed. Human gaits could reflect the walker’s emotional state, and could be an information source for emotion recognition. This paper proposed a novel method to recognize emotional state through human gaits by using Microsoft Kinect, a low-cost, portable, camera-based sensor. Fifty-nine participants’ gaits under neutral state, induced anger and induced happiness were recorded by two Kinect cameras, and the original data were processed through joint selection, coordinate system transformation, sliding window gauss filtering, differential operation, and data segmentation. Features of gait patterns were extracted from 3-dimentional coordinates of 14 main body joints by Fourier transformation and Principal Component Analysis (PCA). The classifiers NaiveBayes, RandomForests, LibSVM and SMO (Sequential Minimal Optimization) were trained and evaluated, and the accuracy of recognizing anger and happiness from neutral state achieved 80.5% and 75.4%. Although the results of distinguishing angry and happiness states were not ideal in current study, it showed the feasibility of automatically recognizing emotional states from gaits, with the characteristics meeting the application requirements.

## Introduction

Emotion is the mental experience with high intensity and high hedonic content (pleasure/displeasure) (Cabanac, 2002), which deeply affects our daily behaviors by regulating individual’s motivation (Lang, Bradley & Cuthbert, 1998), social interaction (Lopes et al., 2005) and cognitive processes (Forgas, 1995). Recognizing other’s emotion and responding adaptively to it is a basis of effective social interaction (Salovey & Mayer, 1990), and since users tend to regard computers as social agents (Pantic & Rothkrantz, 2003), they also expect their affective state being sensed and taken into account while interacting with computers. As the importance of emotional intelligence for successful inter-personal interaction, the computer’s capability of recognizing automatically and responding appropriately to the user’s affective feedback had been confirmed as a crucial facet for natural, efficacious, persuasive and trustworthy human–computer interaction (Cowie et al., 2001; Pantic & Rothkrantz, 2003; Hudlicka, 2003). The possible applications of such an emotion-sensitive system are numerous, including automatic customer services (Fragopanagos & Taylor, 2005), interactive games (Barakova & Lourens, 2010) and smart homes (Silva, Morikawa & Petra, 2012), etc. Although automated emotion recognition is a very challenging task, the development of this technology would be of great value.

As the common use of multiple modalities to recognizing emotional states in human-human interaction, various clues have been used in affective computing, such as facial expressions (e.g., Kenji, 1991), gestures (e.g., Glowinski et al., 2008), physiological signals (e.g., Picard, Vyzas & Healey, 2001), linguistic information (e.g., Alm, Roth & Sproat, 2005) and acoustic features (e.g., Dellaert, Polzin & Waibel, 1996). Beyond those, gait is another modality with great potential. As a most common daily behavior which is easily observed, the body motion and style of walking have been found by psychologists to reflect the walker’s emotional states. Human observers were able to identify different emotions from gait such as the amount of arm swing, stride length and heavy-footedness (Montepare, Goldstein & Clausen, 1987). Even when the gait information was minimized by use of point-light displays, which meant to represent the body motion by only a small number illuminated dots, observers still could make judgments of emotion category and intensity (Atkinson et al., 2004). The attribution of the features of gaits and other body languages to the recognition of specific affective states had been summarized in a review (Kleinsmith & Bianchi-Berthouze, 2013).

In recent years, gait information has already been used in affective computing. Janssen et al. (2008) reported emotion recognition using artificial neural nets in human gait by means of kinetic data collected by force platform and kinematic data captured by motion capture system. With the help of marker-based motion tracking system, researchers developed computing methods to recognize emotions from gait in inter-individual (comparable to recognizing the affective state of an unknown walker) as well as person-dependent status (comparable to recognizing the affective state of a familiar walker) (Karg et al., 2009a; Karg et al., 2009b; Karg, Kuhnlenz & Buss, 2010). These gait information recording technologies had already made it possible to automatically recognize the emotional state of a walker, however, because of the high cost of trained person, technical equipment and maintenance (Loreen, Markus & Bernhard (2013)), the application of these non-portable systems were seriously limited.

The Microsoft Kinect is a low-cost, portable, camera-based sensor system, with the official software development kit (SDK) (Gaukrodger et al., 2013; Stone et al., 2015; Clark et al., 2013). As a marker-free motion capture system, Kinect could continuously monitor three-dimensional body movement patterns, and is a practical option to develop an inexpensive, widely available motion recognition system in human daily walks. The validity of Kinect has been proved in the studies of gesture and motion recognition. Kondori et al. (2011) identified head pose using Kinect, Fern’ndez-Baena, Susin & Lligadas (2012) found it perform well in tracking simple stepping movements, and Auvinet et al. (2015) successfully detected gait cycles in treadmill by Kinect. In Weber et al.’s (2012) report, the accuracy and sensitivity of kinematic measurements obtained from Kinect, such as reaching distance, joint angles, and spatial–temporal gait parameters, were estimated and found to be comparable to gold standard marker-based motion capture systems like Vicon. On the other hand, recently it has also been reported the application of Kinect in the medical field. Lange et al. (2011) used Kinect as a game-based rehabilitation tool for balance training. Yeung et al. (2014) found that Kinect was valid in assessing body sway in clinical settings. Kinect also performed well in measuring some clinically relevant movements of people with Parkinson’s disease (Galna et al., 2014).

Since walkers’ emotional states could be reflected in their gaits (Montepare, Goldstein & Clausen, 1987; Atkinson et al., 2004), and Kinect has been found a low-cost, portable, but valid instrument to record human body movement features (Auvinet et al., 2015; Weber et al., 2012), using Kinect to recognize emotion by gaits could be a feasible practice. Because of the great value of automatic emotion recognition (Cowie et al., 2001; Silva, Morikawa & Petra, 2012), this practice is worth trying. By automating the record and analysis of body expressions, especially the application of machine learning methods, researchers were able to make use of more and more low-level features of configurations directly described by values of 3D coordinate in emotion recognition (De Silva & Bianchi-Berthouze, 2004; Kleinsmith & Bianchi-Berthouze, 2007). The data-driven low-level features extracted from the original 3D coordinates could not provide an intuitive, high-level description of the gaits pattern under certain affective state, but may be used to train computational models effectively recognizing emotions. We hypothesize that the walkers’ emotional states (such as happiness and anger) could be reflected in their gaits information recorded by Kinect in the form of coordinates of the main joints of body, and the states could be recognized through machine learning methods. We conducted an experiment to test this hypothesis and try to develop a computerized method to recognize emotions from Kinect records of gaits.

## Methods

### Experiment design

**Figure 1:**
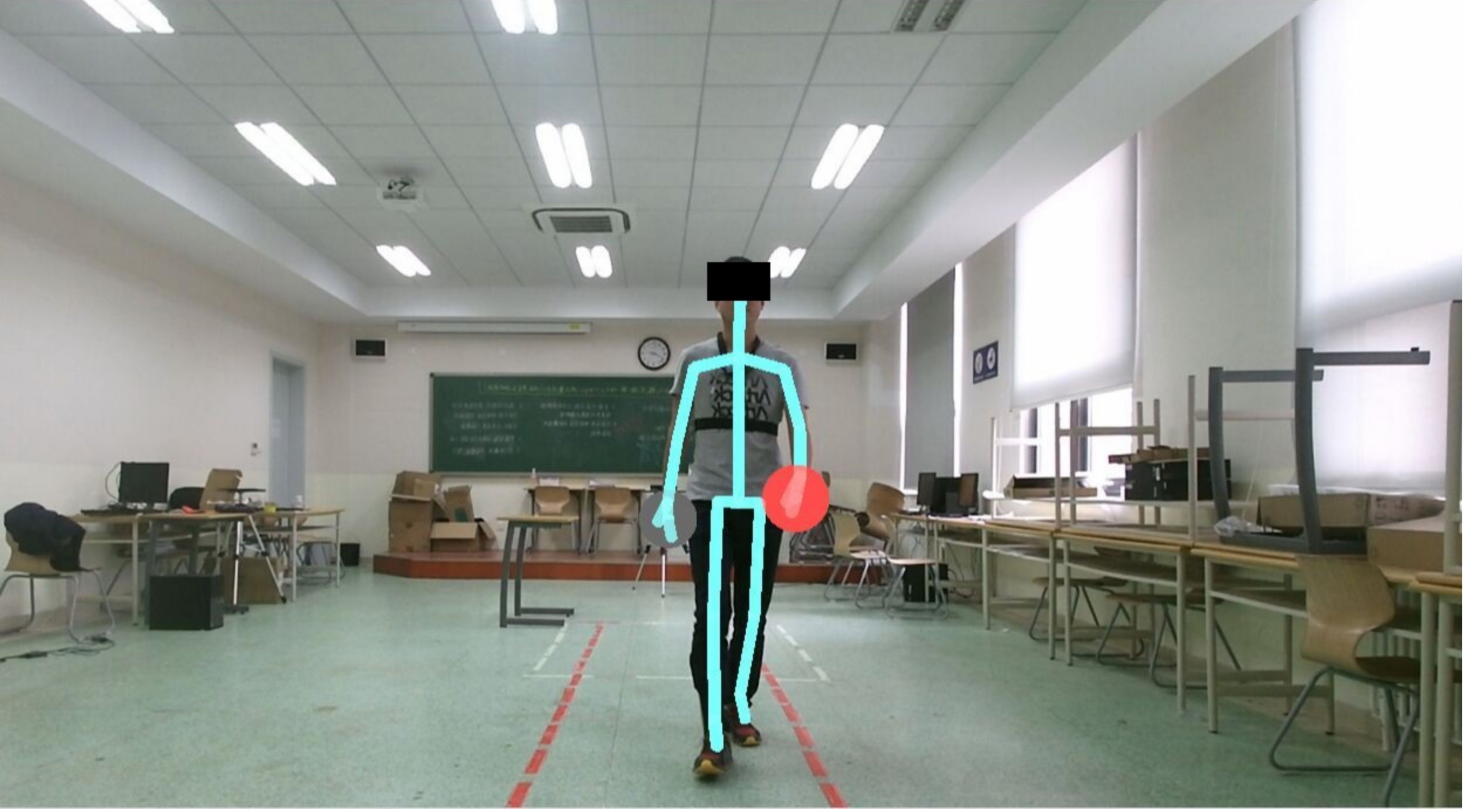
The experiment scene.

**Figure 2:**
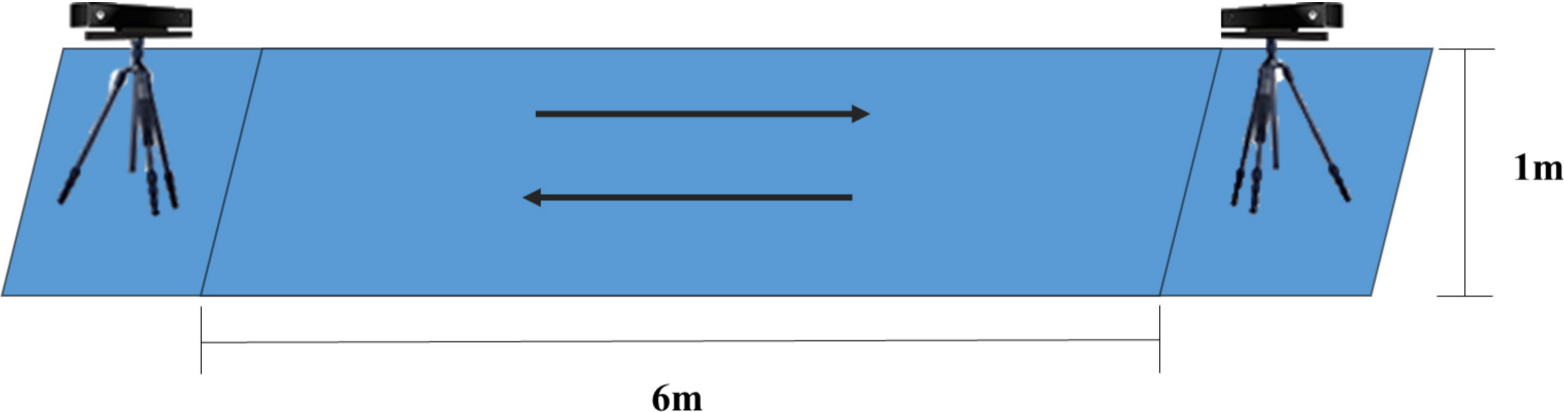
The schematic of the experiment environment.

Fifty-nine graduate students of University of Chinese Academy of Sciences, including 32 females and 27 males with the average age 24.4 (*SD* = 1.6), participated in this study. A good health status based on their self-report was required, and individuals were excluded if they reported any injury or disability affecting walking. The experiment was conducted in a bright, quiet room with a 6 m*1 m footpath marked by adhesive tape on the floor in the center of the room (Fig. 1). Two Kinect cameras were placed oppositely at the two ends of the footpath, to record gaits information (Fig. 2).

**Figure 3:**
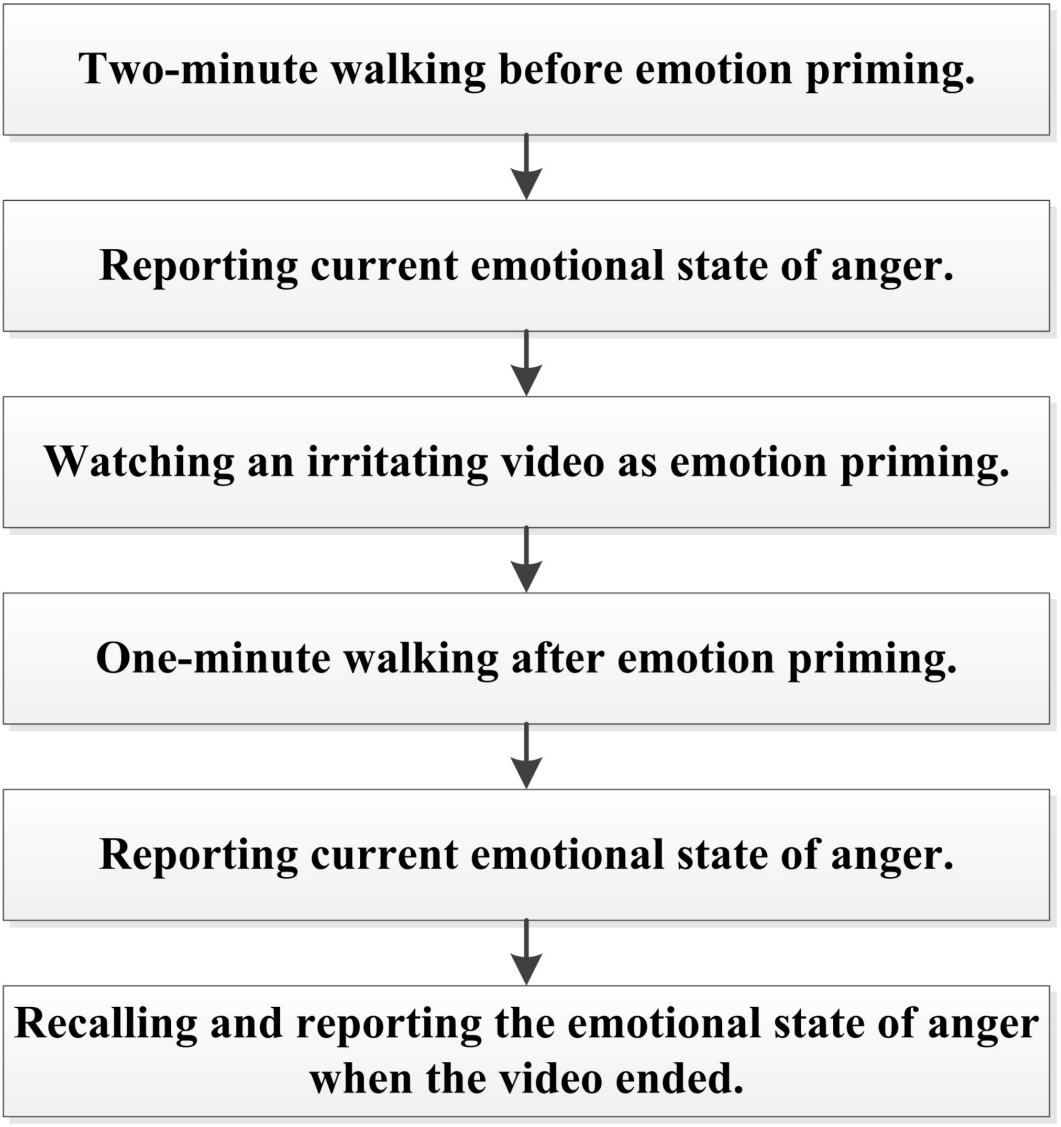
The procedures of the first round experiment.

After informed consent, participants took the first round experiment to produce the gaits under neutral and angry state. Starting from one end of the footpath, participants firstly walked back and forth on the footpath for 2 min (neutral state), while the Kinect cameras recorded their body movements. Then participants were required to mark their current emotional state of anger on a scale from 1 (no anger) to 10 (very angry). Next, participants watched an about 3-minute video clip of an irritating social event, which was selected from a Chinese emotional film clips database and has been used to elicit audience’s anger (Yan et al., 2014), on a computer in the same room. To ensure the emotion aroused by the video lasting during walking, participants started to walk on the footpath back and forth immediately after watching the video (angry state). When this 1-minute walking under induced anger finished, they were asked to mark their current emotional state of anger and their state just when the video ended on the ten-point scale. Figure 3 shows the entire process of this first round experiment.

The second round experiment was conducted following the same procedures, while the video was a funny film clip (Yan et al., 2014) and the scale was measuring the emotional state of happiness. There was at least 3 hours interval between the two rounds of experiments (participants left, and then came back again) in order to avoid possible interference between the two induced emotional states. Every participant finished the two rounds of experiment and left a 1-minite gait record after anger priming (angry state), a 1-minite gait record after happiness priming (happy state), and two 2-minite gait records before emotion priming as the baseline of each round (neutral state). Every time before starting footpath walking, participants were instructed to walk naturally as in their daily life. The whole protocol was approved by the Ethics Committee of Institute of Psychology, Chinese Academy of Sciences (approved number: H15010).

**Figure 4:**
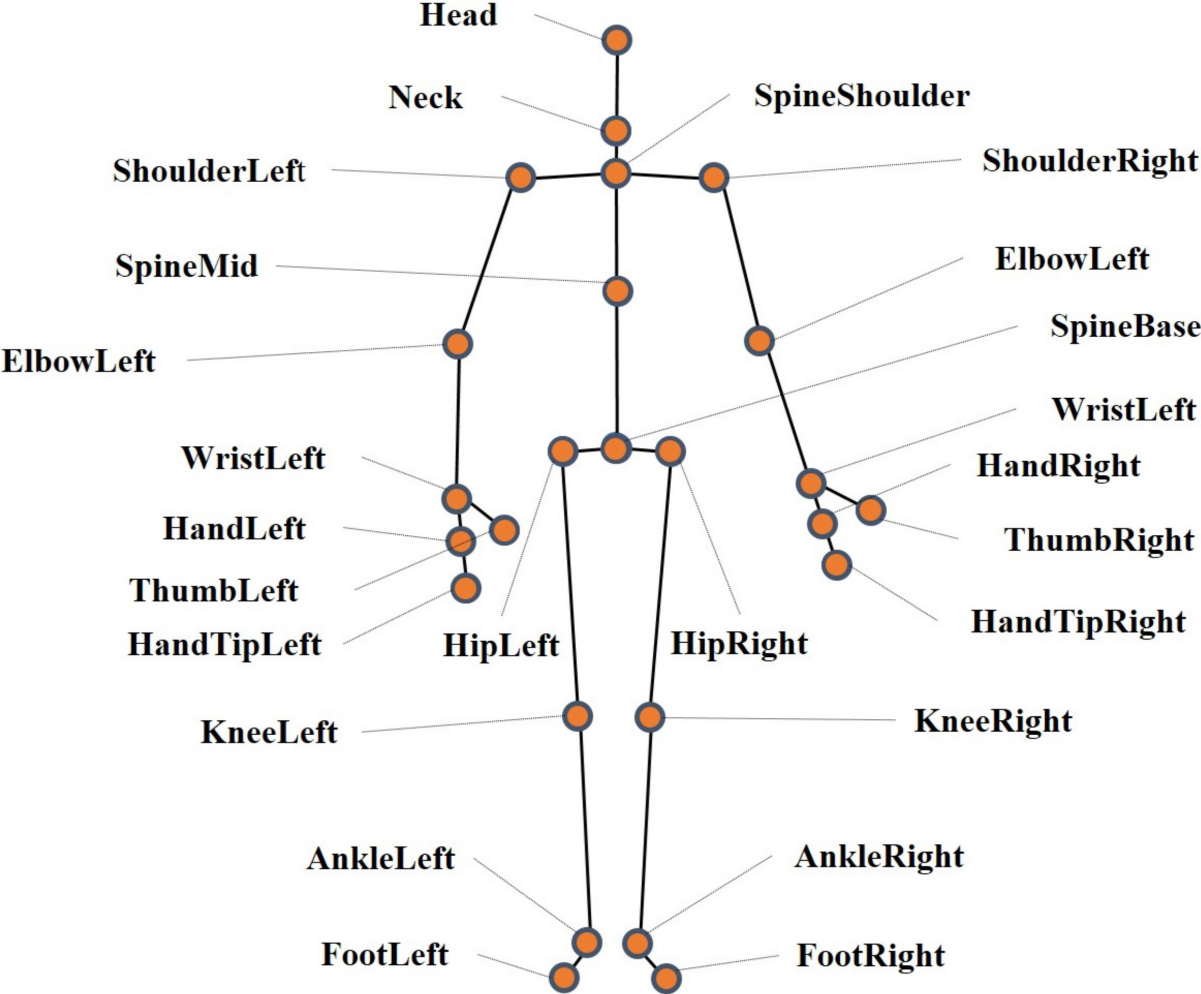
Stick figure and location of body joint centers recorded by Kinect.

### Gaits data collection

With the Kinect cameras placed at the two ends of the footpath, participants’ gaits information was recorded as video on 30 Hz frame rate. Each frame contains 3-dimensional information of 25 joints of body, including head, shoulders, elbows, wrists, hands, spine (shoulder, mid and base), hips, knees, ankles and feet, as shown in Fig. 4. With the help of official Microsoft software development SDK Beta2 of Kinect and customized software (Microsoft Visual Studio 2012), 3-dimensional coordinates of the 25 joints, with the camera position as the origin point, were exported and further processed. The gaits data recorded by the two Kinect cameras were processed independently.

### Data processing

#### Preprocessing

##### Joint selection.

As shown in the psychological studies on the perception of biological movements (e.g., Giese & Poggio, 2003), a few points representing trunk and limbs were enough to provide information for accurate recognition. With the reference of Troje’s virtual marker model (Troje, 2002) which has been used in many psychological studies, and the principle of simplification, we chose 14 joints to analyze the gait patterns, including spinebase, neck, shoulders, wrists, elbows, hips, knees, and ankles. The spinebase joint was also used to reflect subject’s position on the footpath relative to Kinect, and for coordinate system transformation. After joint selection, one frame contains the 3-dimmension position of the 14 joints, which can affords a 42 dimension vector (see Appendix ‘The description of one frame data’). Each participant left 4 uninterrupted gait records (before anger priming, after anger priming, before happiness priming, and after happiness priming), supposing that each record consisted of T frames, then the data of one record can be described by a *T*∗42 matrix (see Appendix ‘The description of the data of one record’).

##### Coordinate system transformation.

Different subjects may have different positions relative to Kinect camera when they walked on the footpath, and it might cause much error in gait pattern analysis if using 3D coordinates directly with the camera position as the origin point. To address this issue, we changed the coordinate system by using the position of spinebase joint in each frame as the origin point instead (see Appendix ‘The process of coordinate system transformation’).

##### Sliding window gauss filtering.

The original recordings of gaits contained noises and burrs, and needed to be smoothed. We apply sliding window gauss filtering to each column of the matrix of each record. The length of the window is 5 and the convolution kernel *c* = [1, 4, 6, 4, 1]∕16, which is a frequently-used low pass gauss filter (Gwosdek et al., 2012) (see Appendix ‘The process of sliding window gauss filtering’).

##### Differential operation.

Since the change of joints’ position between each frame reflects dynamic part of gaits more than the joints’ position itself, we applied the differential operation on the matrix of each record to obtain the changes of 3-dimension position of 14 joints between each frame (see Appendix ‘The process of differential operation’).

##### Data segmentation.

Since the joints coordinates acquired by Kinect was not accurate while the participant was turning around. We dropped the frames of turning and divided one record into several straight-walking segments. The front segments recorded the gaits when participants faced to the camera, and the back segments recorded the gaits when participants back to the camera. To ensure each segment covered at least one stride, we only kept the segments containing at least 40 frames (see Appendix ‘The process of data segmentation’).

#### Feature extraction

It looks quite different for the front and back of the same gait, so we extracted features from front and back segments separately. Since human walking is periodic and each segment in our study covered at least one stride, we ran Fourier transformation and extracted 42 main frequencies and 42 corresponding phases on each segment. Then the averages of front segments and the averages of back segments of a single record were calculated separately, obtaining 84 features from front segments and 84 features from back features. That is we extracted 168 features from one record totally (see Appendix ‘The process of feature extraction’).

Since the value of different features varied considerably, in case some important features with small values might be ignored while training the model, all the features were firstly processed by Z-score normalization. In order to improve calculation efficiency and reduce redundant information, Principal Component Analysis (PCA) was then utilized for feature selection, as it had been found that PCA could perform much better than other techniques on training sets with small size (Martinez & Kak, 2001). The selected features were used in model training.

#### Model training

Three computational models were established in this process, in order to distinguish anger from neutral state(before anger priming), happiness from neutral state(before happiness priming), and anger from happiness. As different classifiers may result in different classification accuracies for the same data, we trained and evaluated several usually effective classifiers, including NaiveBayes, Random Forests, LibSVM and SMO, to select a better model. These four classification methods were utilized with 10-fold cross-validation. More detailed description of the training process could be seen in Li et al.’s (2014) report.

**Table 1:** Self-report emotional states before and after emotion priming.

	Before priming(BP)	After priming I (API:before walking)	After priming II (APII:after walking)
Round 1: anger priming	1.44(.93)	6.46(1.99)	5.08(1.98)
Round 2: happiness priming	3.88(2.49)	6.61(2.22)	5.63(2.24)

**Notes.**

The average of participants’ self-ratings was shown in the table with the standard deviation in the parenthesis.

**Table 2:** The accuracy of recognizing angry and neutral.

	NaiveBayes	RandomForests	LibSVM	SMO
KINECT1	80.5085	52.5424	72.0339	52.5424
KINECT2	75.4237	−	71.1864	−

**Notes.**

Table entries are accuracies expressed as a percentage. Values below chance level (50%) are not presented.

## Results

### Self reports of emotional states

In the current study, self-report emotional states on 10 point scales were used to estimate the effect of emotion priming. As shown in Table 1, for both anger and happiness priming, the emotional state ratings of AP(After priming)I and APII were higher than BP(Before priming). Paired-Samples *t* Test (by SPSS 15.0) showed that: for anger priming, anger ratings before priming was significantly lower than API (*t*[58] = 18.98, *p* < .001) and APII (*t*[58] = 14.52, *p* < .001); for happiness priming, happiness ratings before priming was also significantly lower than API (*t*[58] = 10.31, *p* < .001) and APII (*t*[58] = 7.99, *p* < .001). These results indicated that both anger and happiness priming were successfully eliciting changes of emotional state on the corresponding dimension. In the first round experiment participants were generally experiencing more anger while walking after video than before video, and the same happened for happiness in the second round experiment.

**Table 3:** The accuracy of recognizing happy and neutral.

	NaiveBayes	RandomForests	LibSVM	SMO
KINECT1	79.6610	51.6949	77.9661	−
KINECT2	61.8644	51.6949	52.5414	−

**Notes.**

Table entries are accuracies expressed as a percentage. Values below chance level (50%) are not presented.

**Table 4:** The accuracy of recognizing angry and happy.

	NaiveBayes	RandomForests	LibSVM	SMO
KINECT1	52.5424	55.0847	−	51.6949
KINECT2	−	51.6949	−	50.8475

**Notes.**

Table entries are accuracies expressed as a percentage. Values below chance level (50%) are not presented.

### The recognition of primed emotional states and neutral state

The accuracy of a classifier was the proportion of the correctly classified cases in the test set. Table 2 shows the accuracy of each classifier in recognizing angry and neutral. The results from the data captured by the two Kinect cameras were presented separately. With the classifiers NaiveBayes and LibSVM, the computational model could recognize anger in a relatively high accuracy, especially for NaiveBayes.

Table 3 shows the accuracy of each classifier in recognizing happy and neutral. NaiveBayes and LibSVM, especially the former, also performed better than the other two classifiers. The accuracy could be up to 80% for both angry state recognition and happy state recognition. There were steady differences between the results of KINECT1 and KINECT2: the accuracy using data recorded by KINECT1 was better than KINECT2.

### The recognition of angry and happy

While the accuracy of recognizing primed states and neutral state was fairly high, the performance of the same classifiers on distinguishing angry state and happy state was not ideal. As shown in Table 4, the highest accuracy, which came from Random Forests, was only around 55%. Generally, it seemed that the recognition accuracies of these computational models in current study were not much above chance level. The results from KINECT1 data also seemed a little better than those from KINECT2.

## Discussion

Our results partly supported the hypothesis presented in the end of Introduction: the walkers’ emotion states (such as happiness and anger) could be reflected in their gaits recorded by Kinect in the form of coordinates of the main joints of body, and the states could be recognized through machine learning methods. Participants’ self-reports indicated that the emotion priming in the current study successfully achieved expected effect: participants did walk under an angrier emotional state than before anger priming, and walk under a happier emotional state than before happiness priming. In fact, the emotional state while walking before priming in the study could be seen as neutral, representing the participant’s “normal state” as we did not exert any influence. So the current results could be seen as distinguishing angry/happy state from neutral state, just by the Kinect-recorded data of gaits.

These results also showed the feasibility of recognizing emotional states by using gaits, with the help of low-cost, portable Kinect cameras. Although some gait characteristics such as the amount of arm swing, stride length and walking speed had been found to reflect the walker’s emotional state (Montepare, Goldstein & Clausen, 1987), it is not surprising that making accurate judges of emotional state automatically based on these isolated indicators could be difficult for computer, and even for human observers if the intensity of the target emotion was slight. The recognizing method in our study did not depend on few certain emotion-relevant indexes of body movements, but made use of continuous dynamic information of gaits in the form of 3D coordinates. Machine learning made it possible to make full use of these low-level features, and the high accuracy of recognition by some common classifiers implied the validity of this method. In fact, the low-level features used in this study did not exclusively belong to walking, and there was great potential to use this method in recognizing emotions by other types of body motions.

However, in the current study we did not distinguish between two primed emotional states, anger and happiness, from each other very well. In principle, there were two possible reasons. First, the anger and happiness elicited in the experiment may be relatively slight and seem to be similar while reflected in gaits. In previous studies, participants were often required to recall a past situation or imagine a situation associated to certain affect while walking (Roether et al., 2009; Gross, Crane & Fredrickson, 2012), and the emotional states induced may be relatively stronger than in our study. As high-arousal emotions, both anger and happiness share some similar kinematic characteristics (Gross, Crane & Fredrickson, 2012), which also makes it difficult to distinguish each other by computational models. Second, it was also possible that the difference between anger and happiness had already been presented in gaits, but the methods of feature extraction model training were not sensitive enough to make use of them. Considering the evidence from other reports on the difference of gait features between angry and happy state (Montepare, Goldstein & Clausen, 1987; Roether et al., 2009; Gross, Crane & Fredrickson, 2012), further optimized computing methods may be valid to distinguish those slight, but different emotional states.

Although the current method could only recognize induced emotions and neutral states, it has the potential to bring benefits in application. The automatically recognized emotional arousal state could be a valuable source for decision making, together with other information. For example, if the emotional arousal was detected from a person who has been diagnosed with Major Depression Disorder, it would probably be an indicator of negative mood episode. Another example could be the practice of security personnel in detecting hostility or deception. In this situation with certain background knowledge, the automatic detection of an unusual state of emotional arousal from the target would be a meaningful cue for the observer mitigating information overload and reducing misjudgments, even when the emotion type of that arousal is not precisely determined. Compared to requiring participants to act walking with certain emotion (Westermann, Stahl & Hesse, 1996), the walking condition of participants in our study was more common in daily life, increasing the possibility of making use of our method. Moreover, one Kinect camera was able to trace as many as six individuals’ body movements, making it convenient to monitor and analyze affects in emotional social interactions among individuals. Since emotional features of mental disorders often appeared in the condition of interpersonal interaction (Premkumar et al., 2013; Premkumar et al., 2015), using Kinect data to recognize affects may be of even more practical value than other methods.

There were still some limitations of this study. There was steady difference of the data quality from the two Kinect cameras, which probably indicated that we did not perfectly control the illumination intensity of our experiment environment. To make the experiment condition as close to natural state as possible, we used the natural emotional state before priming as the baseline without any manipulation, so the baseline was only “approximately” neutral and might be different among participants. Despite those limitations, the present study shows a feasible method of automatically recognizing emotional states from gaits using the portable, low-cost Kinect cameras, with great potential of practical value. It would be worthwhile for future studies to improve this method, in order to raise the efficiency of distinguishing certain types of affect. In our study the extraction of low-level features from gaits was indiscriminate. In fact, it had been found that different facets of gait patterns may relate to certain dimensions of emotion (such as arousal level or valence) in varying degrees (Pollick et al., 2001). So a possible strategy in future study might be adding some features designed based on the characteristics of the target emotion, for better utilization of the features in model training.

##  Supplemental Information

10.7717/peerj.2364/supp-1 Supplemental Information 1 59 subjects’ gait patterns in two-round experiments on two Kinects

## Appendix. The preprocessing and feature extraction of data

### The description of one frame data

One frame gait data, after selecting 14 joints, can be expressed by a 42 dimension vector:

(1) \begin{eqnarray*}{j}_{t}=[{x}_{1},{y}_{1},{z}_{1},{x}_{2},{y}_{2},{z}_{2},\ldots ,{x}_{14},{y}_{14},{z}_{14}].\end{eqnarray*}

### The description of the data of one record

Suppose that the data of one record consisted of *T* frames. One record could be expressed by the *T*∗42 matrix, with *j*_1_, *j*_2_, ..., *j*_*t*_, ..., *j*_*T*_ as 42 dimension vectors describing each frame:

(2) \begin{eqnarray*}J=[{j}_{1},{j}_{2},\ldots ,{j}_{t},\ldots ,{j}_{T}]^{T}.\end{eqnarray*}

### The process of coordinate system transformation

In one frame data *j*_*t*_, the first three columns were the 3-dimension coordinates of the spinebase joint, so the coordinate transformation was conducted by:

(3) \begin{eqnarray*}\begin{array}{@{}l@{}} \displaystyle {x}_{i}^{t}={x}_{i}^{t}-{x}_{1}^{t}\\ \displaystyle {y}_{i}^{t}={y}_{i}^{t}-{y}_{1}^{t}\\ \displaystyle {z}_{i}^{t}={z}_{i}^{t}-{z}_{1}^{t}\\ \displaystyle (1\leq t\leq T,2\leq i\leq 14). \end{array}\end{eqnarray*}

### The process of sliding window gauss filtering

The process of filtering was conducted as follow:

(4) \begin{eqnarray*}\begin{array}{@{}l@{}} \displaystyle {x}_{i}^{t}=[{x}_{i}^{t},{x}_{i}^{t+1},{x}_{i}^{t+2},{x}_{i}^{t+3},{x}_{i}^{t+4}]~\boldsymbol{ \cdot }~c\\ \displaystyle {y}_{i}^{t}=[{y}_{i}^{t},{y}_{i}^{t+1},{y}_{i}^{t+2},{y}_{i}^{t+3},{y}_{i}^{t+4}]~\boldsymbol{ \cdot }~c\\ \displaystyle {z}_{i}^{t}=[{z}_{i}^{t},{z}_{i}^{t+1},{z}_{i}^{t+2},{z}_{i}^{t+3},{z}_{i}^{t+4}]~\boldsymbol{ \cdot }~c\\ \displaystyle (1\leq t\leq T-4,1\leq i\leq 14). \end{array}\end{eqnarray*}

### The process of differential operation

To obtain the changes of 3-dimension position of 14 joints between each frame, the differential operation is conducted by:

(5) \begin{eqnarray*}{j}_{t-1}={j}_{t}-{j}_{t-1}(2\leq t\leq T).\end{eqnarray*}

### The process of data segmentation

One record was divided into several front segments and back segments. If one record *J* contained *n* front segments and *m* back segments, it could be described as a series of matrices as follow. Each matrix had 42 columns, but may have different counts of rows, because each segment may contain different number of frames:

(6) \begin{eqnarray*} \left\{ \begin{array}{@{}l@{}} \displaystyle {Front}_{i},1\leq i\leq n\\ \displaystyle {Back}_{j},1\leq j\leq m. \end{array} \right. \end{eqnarray*}

### The process of feature extraction

We ran Fourier transformation to acquire features of each *Front*_*i*_ and *Back*_*i*_, obtaining the main frequencies ${f}_{1}^{i},{f}_{2}^{i},\ldots ,{f}_{42}^{i}$ and the corresponding phases ${\varphi }_{1}^{i},{\varphi }_{2}^{i},\ldots ,{\varphi }_{42}^{i}$ for each segment. The averages of *n* front segments and *m* back segments of a single record were calculated separately, obtaining *Feature*_*front*_ and *Feature*_*back*_ of each record:

(7) \begin{eqnarray*}{Feature}_{front}= \frac{1}{n} \sum _{i}^{n}[{f}_{1}^{i},{f}_{2}^{i},\ldots ,{f}_{42}^{i},{\varphi }_{1}^{i},{\varphi }_{2}^{i},\ldots ,{\varphi }_{42}^{i}]\end{eqnarray*}

(8) \begin{eqnarray*}{Feature}_{back}= \frac{1}{m} \sum _{i}^{m}[{f}_{1}^{i},{f}_{2}^{i},\ldots ,{f}_{42}^{i},{\varphi }_{1}^{i},{\varphi }_{2}^{i},\ldots ,{\varphi }_{42}^{i}].\end{eqnarray*}

*Feature*_*front*_ and *Feature*_*back*_ of each record contained 84 features respectively, and the complete feature matrix *Feature* of each record was the combination of *Feature*_*front*_ and *Feature*_*back*_, containing 168 features:

(9) \begin{eqnarray*}Feature=[{Feature}_{front},{Feature}_{back}].\end{eqnarray*}

## Additional Information and Declarations

### Data Availability

The following information was supplied regarding data availability: The raw data has been supplied as a Supplemental Dataset.
